# *Piscirickettsia salmonis* Produces a N-Acetyl-L-Homoserine Lactone as a Bacterial Quorum Sensing System-Related Molecule

**DOI:** 10.3389/fcimb.2021.755496

**Published:** 2021-10-25

**Authors:** Pamela Ruiz, Daniela Sepulveda, José Miguel Vidal, Romina Romero, David Contreras, Javier Barros, Carlos Carrasco, Nathaly Ruiz-Tagle, Alex Romero, Homero Urrutia, Cristian Oliver

**Affiliations:** ^1^ Laboratorio de Biopelículas y Microbiología Ambiental, Centro de Biotecnología, Universidad de Concepción, Concepción, Chile; ^2^ Departamento de Ciencias Biológicas, Facultad de Ciencias de la Vida, Universidad Andres Bello, Talcahuano, Chile; ^3^ Departamento de Investigación y Desarrollo, Ecombio limitada, Concepción, Chile; ^4^ Laboratorio de Investigaciones Medioambientales de Zonas Áridas (LIMZA), Depto. Ingeniería Mecánica, Facultad de Ingeniería, Universidad de Tarapacá, Arica, Chile; ^5^ Departamento de Química Analítica e Inorgánica, Facultad de Ciencias Químicas, Universidad de Concepción, Concepción, Chile; ^6^ Departamento de Investigación y Desarrollo, Micbiotech Spa, Concepción, Chile; ^7^ Laboratorio de Inmunología y Estrés de Organismos Acuáticos, Facultad de Ciencias Veterinarias, Instituto de Patología Animal, Valdivia, Chile; ^8^ Interdisciplinary Center for Aquaculture Research (INCAR), Concepción, Chile; ^9^ Departamento de Microbiología, Facultad de Ciencias Biológicas, Universidad de Concepción, Concepción, Chile

**Keywords:** *Piscirickettsia salmonis*, SRS, quorum sensing (QS), N-acetyl-L-homoserine lactone, AHL (N-acyl-homoserine lactone)

## Abstract

*Piscirickettsia salmonis* is the etiological agent of piscirickettsiosis, the most prevalent disease in salmonid species in Chilean salmonids farms. Many bacteria produce *N*-acyl-homoserine lactones (AHLs) as a quorum-sensing signal molecule to regulate gene expression in a cell density-dependent manner, and thus modulate physiological characteristics and several bacterial mechanisms. In this study, a fluorescent biosensor system method and gas chromatography-tandem mass spectrometry (GC/MS) were combined to detect AHLs produced by *P. salmonis.* These analyses revealed an emitted fluorescence signal when the biosensor *P. putida* EL106 (RPL4cep) was co-cultured with both, *P. salmonis* LF-89 type strain and an EM-90-like strain Ps007, respectively. Furthermore, the production of an AHL-type molecule was confirmed by GC/MS by both *P. salmonis* strains, which identified the presence of a N-acetyl-L-homoserine Lactone in the supernatant extract. However, It is suggested that an alternate pathway could synthesizes AHLs, which should be address in future experiments in order to elucidate this important bacterial process. To the best of our knowledge, the present report is the first to describe the type of AHLs produced by *P. salmonis*.

## Introduction

Piscirickettsiosis is the main infectious disease affecting Chilean aquaculture (Figueroa et al., 2019). This disease produces high mortality in Atlantic salmon (*Salmo salar*), Coho salmon (*Oncorhynchus kisutch*) and rainbow trout (*Oncorhynchus mykiss*) and causes serious economic losses (Rozas and Enríquez, 2014). Piscirickettsiosis is a systemic disease generated by *Piscirickettsia salmonis*, a Gram-negative facultative intracellular γ-proteobacterium, which is able to evade the host immune response and establishes a replicative vacuole in the cytoplasm (Ortiz-Severín et al., 2019; Pérez-Stuardo et al., 2020). Current prevention strategies for fish farmed in seawater are based on vaccination; of the 54 registered fish vaccines in Chile, 34 are for Piscirickettsiosis (SAG, 2021). However, protection by vaccination has been difficult to achieve in the long term under field conditions (Figueroa et al., 2019). Additionally, In addition, antibiotics treatments used in aquaculture have not been completely effective in reducing the development of salmonid rickettsial septicemia (SRS) (Miranda et al., 2018), probably due to antibiotic resistance acquired by the pathogen (Henríquez et al., 2016; Sandoval et al., 2016; Cartes et al., 2017) and SRS outbreaks are recurrent in Chile (Sernapesca, 2021). Several studies have described the intracellular life cycle of *P. salmonis* (McCarthy et al., 2008; Rojas et al., 2009; Pérez-Stuardo et al., 2019; Zúñiga et al., 2020) and even the biofilm formation as a survival strategy of *P. salmonis* in the seawater (Marshall et al., 2012; Levipan et al., 2020). However, the molecular mechanisms of *P. salmonis* communication its responses to specific external stimuli are largely unknown.

Bacteria use Quorum Sensing (QS)-mediated communication systems to coordinate physiological functions such as DNA uptake, biofilm formation, defense strategies and secretion of virulence factors (Duddy and Bassler, 2021; Wu and Luo, 2021). The expression of virulence genes *via* QS includes I- the synthesis of QS signal molecules; II- the release of signaling molecules; III- sensing and binding of the signaling molecules to membrane receptors at high cell density; IV- the retrieval of the receptor-signal complex from the cell and its binding to the promoter region; and V- the transcription of pathogenicity-related genes (Deng et al., 2011; Durán et al., 2016). However, the question of whether the expression of these virulence factors is or not regulated by a QS system in *P. salmonis* has not yet been studied.

Many γ-proteobacteria orchestrate their behavior in a cell density-dependent manner *via* N-acyl-homoserine lactones (AHLs) (Miller and Bassler, 2001), which consist of a polar homoserine lactone head group and a nonpolar aliphatic tail (Fuqua and Greenberg, 2002; Whiteley et al., 2017). Most of the described AHLs are fatty acyl AHLs with acyl groups of various lengths (C_4_ to C_18_). In addition, an AHL molecule containing a C_2_- acyl group has recently been reported in *Gluconacetobacter* strains (Liu et al., 2019). AHLs possess either a hydroxyl, carbonyl, or no substitution on the third carbon and have various degrees of side-chain saturation (Whitehead et al., 2001; Waters and Bassler, 2005). A typical AHL-QS system contains an AHL signal synthase (LuxI) and a transcriptional regulator (LuxR) (Tsai and Winans, 2010; Papenfort and Bassler, 2016). For example, QS in *P. aeruginosa* is regulated by N-3-oxo-dodecanoyl L-homoserine lactone (3-oxo-C12-AHL) and N-butyryl L-homoserine lactone (C4-AHL), which bind to intracellular LuxR-type receptors and induce the expression of LuxI-type AHL synthases (Brotherton et al., 2018). However, bacteria can contain multiple *luxI-luxR* homologs and possess orphan or “solo” *luxR* homologs (Hudaiberdiev et al., 2015). Therefore, many bacteria also contain extra copies of *luxR* transcriptional regulators that are not proximal to a *luxI* synthase gene (Gan et al., 2015). *LuxI* and *luxR* homologs have been identified in diverse species throughout the Gram-negative Proteobacteria, including phototrophic purple non-sulfur bacteria, marine *Vibrios*, rhizosphere bacteria, enteric commensals and opportunistic pathogens of plants and animals (Parsek and Greenberg, 2000; Gray and Garey, 2001). It has been reported that strains of *P. salmonis* have between 2 and 5 copies of *luxR* (Millar et al., 2018), and LuxR is present in the outer membrane vesicles of *P. salmonis* LF-89 type strain (Oliver et al., 2017). However, there is no evidence of synthase *LuxI* gene orthologs or *luxI*-like genes in *P. salmonis.*

Recently, Levipán et al. (2021) reported that *P. salmonis* does not possess a functional QS circuit based on the endogenous production of AHL-type molecules. However, the authors suggest *P. salmonis* could have a functional QS system in the presence of exogenous AHL-type molecules. Little is known about AHLs-type molecules production in marine pathogens, and AHL production by *P. salmonis* has not been conclusively documented. However, fluorescence-based biosensors have advantages over other methods due to the fluorescence intensity of single cells can be measured (Rai et al., 2015). Therefore, the selection of the most appropriate fluorescent biosensor bacterium for *P. salmonis* is important in order to optimize the detection of fluorescence induced by AHL-like molecules. We aimed to determine if *P. salmonis* can produce active QS AHLs-type molecules by using green fluorescent protein-based biosensor strains and if so, to identify the types of these molecules by Gas-chromatography coupled to mass spectrometry.

## Material and Methods

### Bacterial Culture

*P. salmonis* LF-89 (equivalent to ATCC VR-1361) type strain and an EM-90-like isolate (Ps007) were routinely grown on AUSTRAL-TSFe agar plates at 18°C for ten days (Yáñez et al., 2013). For all experiments, *P. salmonis* colonies were grown in AUSTRAL-SRS broth (ASB) at 18°C for 120 h and 75 rpm (Yañez et al., 2012). The identity of each isolate was confirmed by phenotyping procedures and PCR assays (Karatas et al., 2008; Isla et al., 2021). Bacterial cell density was measured using the BacLight™ LIVE/DEAD Viability Kit (Invitrogen™, USA). To verify the purity of *P. salmonis* in all the tests performed, before and after the cultures, direct microscopy methods were carried out like Gram staining and/or epifluorescence microscopy using the BacLight™ LIVE/DEAD Viability Kit (SYTO 9/propidium Iodide) (Invitrogen™, USA). In parallel, an aliquot was transferred onto tryptone soy agar plates supplemented with hemoglobin (Austral-TSHem) at 18°C for 10-15 days to validate the purity results by observing the growth of only *P. salmonis* colonies, and additional Gram staining procedures.

### Selection of Bacterial Biosensor for AHLs Detection

The biosensor with the highest sensitivity for N-Octanoyl-L-homoserine lactone (C8-HSL) molecules was selected by testing the *Pseudomonas putida* EL105 (RPL4las), *P. putida* EL106 (RPL4cep) and *Escherichia coli* MT102 (pKR-C12) biosensor strains (Lumjiaktase et al., 2010). Bacterial strains were previously grown overnight at 27°C in Luria Bertani (LB Miller, Merck) agar plates with Kanamycin (100 μg/mL, Sigma-Aldrich, St. Louis, MO) and Rifampicin (50 μg/mL, Sigma-Aldrich, St. Louis, MO) for *Pseudomonas* strains and only Gentamicin (25 μg/mL, US Biological) for *E. coli*. Then, single colonies of each strain were inoculated in 25 mL of LB broth (Miller, Merck) with antibiotics and incubated for 12 h as described above. Bacterial cells were then resuspended and washed three times in milli-Q water by centrifugation at 8,000 rpm for 15 min at 4°C. The cell pellet was resuspended in LB broth with four μM C8-HSL (HPLC-grade; Sigma-Aldrich, St. Louis, MO) at a 1x10^9^ cells/mL final density and then incubated for 24 h at 27°C and 120 rpm as previously described (Lumjiaktase et al., 2010). After that, *P. putida* EL106 (RPL4cep) was grown in the presence of C8-HSL in a concentration range from 10 nM to 1600 nM in order to determine the optimal concentration to use in the experiments. Bacterial cells grown without the addition of AHLs in the medium were used as controls. Fluorescence was measured in 96-wells black plates at 475 nm excitation wavelength and 515 nm emission detection in a microplate reader (TECAN infinite, F200 pro). Measurements were done in triplicate, and green fluorescence values were normalized to 1 mL of bacterial culture and converted into specific green fluorescence by dividing normalized values by the OD_600_ of the bacterial culture. Data was obtained using i-control™ Microplate Reader software. Subsequently, the biosensor bacterial strain that showed significantly highest fluorescence was also evaluated at 18°C in the same conditions as previously described to evaluate the successful growth and fluorescence emission of biosensor bacterial strains in subsequent assays with *P. salmonis.* The fluorescence measurements were corrected for autofluorescence.

### Induction of AHLs Biosensor by *P. salmonis* Strains

*P. putida* EL106 (RPL4cep) strain was grown in LB broth with the corresponding antibiotic. Bacterial cells were pelleted and washed three times in milli-Q water by centrifugation at 8,000 rpm for 15 min and 4°C. Subsequently, *P. salmonis* LF-89 and Ps007 strains were grown in ASB for 120 h at 18°C and 75 rpm. After that, the bacterial biosensor was resuspended in ASB at 10^5^ and 10^9^ cells/mL and co-cultured with 10^9^ cells/mL of each *P. salmonis* strain. Cultures were incubated for 24, 96, 144 and 168 h at 18°C and 75 rpm. The bacterial biosensor was resuspended in ASB with 250 nM C8-HSL was used as an inductor response control. This concentration was the highest concentration, within the lowest range of HSL-C8 concentrations used (10 to 250 nM), that emitted the highest fluorescence values, allowing us to optimize the use of the pure inductor and be able to quantify the emitted fluorescence. Fluorescence was measured in triplicate in 96-wells black plates in a microplate reader (TECAN infinite, F200 pro) set at an excitation wavelength of 475 nm and emission detection at 515 nm. Data were obtained using i-control ™ Microplate Reader software. The fluorescence measurements were corrected for autofluorescence.

### Extraction of AHLs From *P. salmonis* Culture Supernatants

AHLs from *P. salmonis* LF-89 and Ps007 strains culture supernatants were obtained according to the method described by (Shaw et al., 1997) with some modifications. Briefly, each *P. salmonis* strain was grown in 600 mL of ASB at 18°C for 120 h and 75 rpm. Bacterial cells were centrifuged at 5,000 rpm for 15 min at 4°C, and the supernatant was extracted three times with a ratio of 1:1 of acidified ethyl acetate (0.5% glacial acetic acid). The organic phase obtained was dried with anhydrous MgSO_4_ (Merck, Darmstadt, Germany), filtered through a cellulose filter (47 mm, 0.22 µm) and concentrated by rotary evaporation at 43 ± 2°C. The dry extract was weighed and resuspended in 5 mL of acetone (GC-ECD/FID grade, Merck, Darmstadt, Germany). The extracts were stored at -20°C until use.

### Induction of AHLs Biosensor by *P. salmonis* Supernatant and Organic Extracts

The filtered supernatant and organic extracts (dissolved in 10% w/v ASB) of each *P. salmonis* strain were arranged in a 1:1 ratio with the bacterial biosensor *P. putida* EL106 (RPL4cep) (10^9^ cells/mL), and the emitted fluorescence was evaluated. RFU was measured at 24 and 48 h in triplicate in 96-wells black plates as described above. The fluorescence measurements were corrected for autofluorescence.

### Identification of AHLs by GC/MS

The identification of AHLs was performed by GC/MS, similar to Levipan et al. (2021). Briefly, the standard solutions of N-butyryl-DLHomoserine lactone (C4-HSL), N-hexanoyl-DL-homoserine lactone (C6-HSL), N-octanoyl-L-homoserine (C8-HSL), and N-dodecanoyl-L-Homoserine lactone (C12-HSL) (HPLC-grade; Sigma-Aldrich, St. Louis, MO) were prepared in GC-ECD/FID-grade acetone. Analysis was performed using a model 7890A GC system (Agilent, USA) interfaced to a single quadrupole mass selective detector 5975C (triple-axis detector), both of which were controlled by a computer equipped with ChemStation software (Agilent, USA) and an internal NIST Mass Spectral Search Program (NIST v.02, 2009). Sample injection was done in splitless mode into an HP-5MS capillary column, 30 m x 250 mm i.d. and 0.25 mm film thickness (Agilent, USA). Pure helium (99.999%, Linde, Chile) was used as the GC carrier gas at a flow rate of 1.0 mL min^-1^. The GC injector temperature was set at 250°C. The GC column-oven temperature was set at 60°C holding for 2 min, then up to 270°C and maintained by 5 min with a heating rate of 10°C min^−1^ (Levipan et al., 2021). One μL of extracted samples and AHLs standards previously diluted with acetone was injected into the GC system for the AHLs identification. The transfer line temperature was adjusted to 280°C. Mass spectrometry conditions were as follows: electron ionization source set to 70 eV, MS Quad 150°C, MS Source 230°C. The mass spectrometer was run in full-scan mode (m/z 15–350) and Selected Ion Monitoring (SIM) at m/z 143.

### Statistical Analysis

Graphpad Prism v7 (GraphPad Software Inc., La Jolla, CA, USA) was used for dataset analysis and graphing of all experiments. Differences between experimental conditions were deemed statistically significant at P ≤ 0.01, as established using Student’s t-test and one-way ANOVA with Tukey Multiple Comparison test (Zar, 1999).

## Results

### Screening of the Fluorescent Bacterial Biosensors for the AHLs Detection

**Figure 1:**
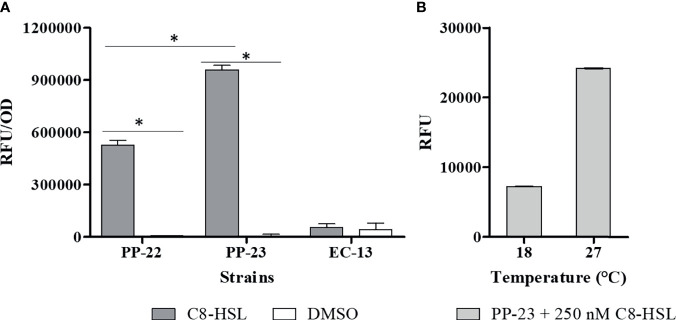
Selection of biosensor strains at the optimal growth conditions of *P. salmonis*. **(A)** Response of 4 μM C8-HSL to biosensor strains; PP-22: *P. putida* EL105 (RPL4las), PP-23: *P. putida* EL106 (RPL4cep), and EC-12: *E. coli* MT102 (pKR-C12) at 27°C for 24 h. Control: DMSO (C8-HSL solvent). **(B)** Response of 250 nM C8-HSL to *P. putida* EL 106 (RPL4cep) at 18°C and 27°C for 24 h. Emitted fluorescence was recorded in a microtiter plate reader at 515 nm. RFU, relative fluorescence units. Asterisks indicate statistical significance (Student’s t-test P ≤ 0.01, n = 3). Error bars: standard deviation.

The emitted fluorescence of each biosensor strain at different culture times was determined in order to select the strain with the highest sensitivity for AHLs. Bacterial biosensors were grown in the presence of C8-HSL in a concentration range from 10 nM to 1600 nM. Bacterial cells grown without the addition of AHLs in the medium were used as controls. Thus, C8-HSL at a concentration of 250 nM was selected for the assay (**Supplementary Figure 1**). We determined that the solvent control (DMSO) for *E. coli* MT102 (pKR - C12) produced high autofluorescence that prevented the detection of C8-HSL. The emitted fluorescence of *P. putida* EL105 (RPL4las) and *P. putida* EL106 (RPL4cep) was significantly (ANOVA, P ≤ 0.01) higher than the control (**Figure 1A**). Importantly, the emitted fluorescence by *P. putida* EL106 (RPL4cep) was significantly (ANOVA, P ≤ 0.01) higher than *P. putida* EL105 (RPL4las). The emitted fluorescence by *P. putida* EL106 (RPL4cep) in the presence of 250 nM AHL-C8 at 18°C was 7232 ± 27.2 RFU, which was 70% lower than the fluorescence at 27°C (24158 ± 80.3 RFU) (**Figure 1B**), indicating it was completely functional at the lowest temperature tested and could be used to evaluate the production of AHLs by *P. salmonis.*

### *P. salmonis* Strains Induce an AHL Response in the Bacterial Biosensor

**Figure 2:**
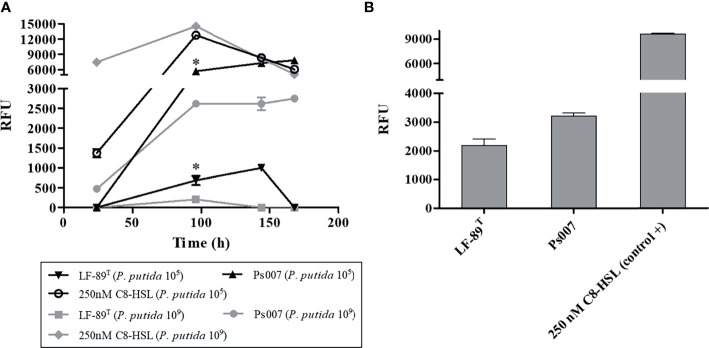
Fluorescence induction in *P. putida* EL 106 (RPL4cep) in co-culture with *P. salmonis* LF-89^T^ and EM-90-like (Ps007) strains. **(A)** *P. putida* EL 106 (RPL4cep) 10^5^ cell/mL and 10^9^ cell/mL at 24, 96, 144 and 168h. Asterisks indicate statistical significance (ANOVA P ≤ 0.01, n = 3) in *P. salmonis* Ps007 strain between 10^5^ and 10^9^ cells/mL of the biosensor strain from 96 h to 168 h. In *P. salmonis* LF-89 strain asterisks indicate statistical significance (ANOVA P ≤ 0.01, n = 3) between 10^5^ and 10^9^ cells/mL of the biosensor strain only at 96 h. **(B)** *P. putida* EL 106 (RPL4cep) 10^5^ cell/mL at 120 h. Control: *P. putida* EL106 (RPL4cep) + 250 nM C8-HSL. Emitted fluorescence was recorded in a microtiter plate reader at 515 nm. RFU, relative fluorescence units. Asterisks indicate statistical significance (ANOVA P ≤ 0.01, n = 3) with the control. Error bars: standard deviation.

The fluorescence emitted by the selected biosensor *P. putida* EL106 (RPL4cep) was evaluated in a co-culture with both *P. salmonis* strains at different concentrations and times. The fluorescent intensity of *P. putida* EL106 (RPL4cep) was higher in the presence of the two *P. salmonis* strains and the positive control at 96 h (**Figure 2A**) at 10^9^ cells/mL. Additionally, the intensity of the fluorescence emitted by the bacterial biosensor increased up to 144 h and 168 h in the presence of the *P. salmonis* LF-89 and Ps007, respectively, at 10^5^ cell/mL concentration. However, this was also the case up to 96 h in the positive control. Ps007 showed the highest fluorescence intensity in both conditions at 168 h, reaching a maximum of 7825 ± 131 RFU and 2750 ± 79 RFU at a biosensor concentration of 10^9^ CFU/mL and 10^5^ CFU/mL, respectively. LF-89 generated the lowest maximum fluorescence intensity of 203 ± 4 RFU and 997 ± 117 RFU at a biosensor concentration of 10^9^ CFU/mL and 10^5^ CFU/mL, respectively. Based on these results, we decided to use a biosensor concentration of 10^5^ cells/mL and a time of 120 h for our next assay. *P. salmonis* LF-89 type strain showed an increase in fluorescence intensity (RFU = 2183 ± 380) under these conditions, while the intensity of *P. salmonis* Ps007 strain was 3209 ± 176 RFU (**Figure 2B**).

### *P. salmonis* Supernatant and Organic Extracts Induce an AHL Response in the Bacterial Biosensor

**Figure 3:**
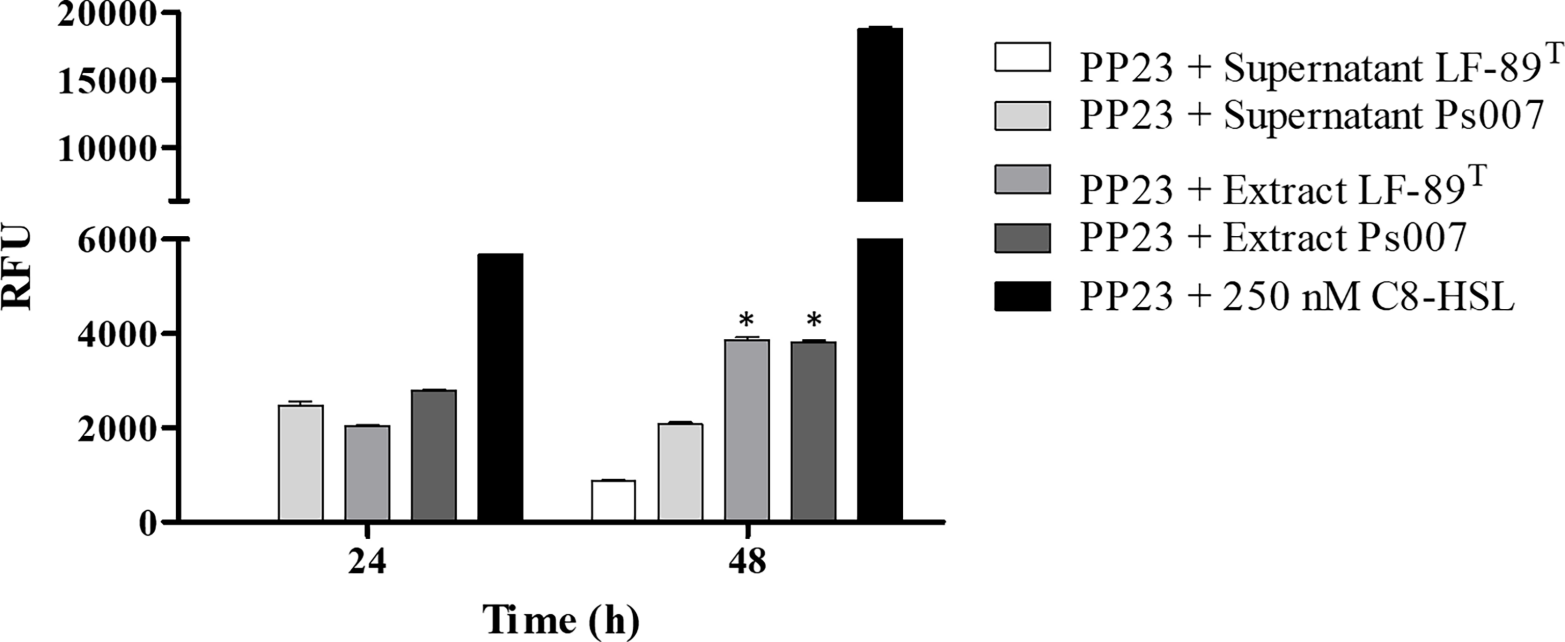
Fluorescence induction in *P. putida* EL 106 (RPL4cep) of supernatants and organic extracts of *P. salmonis* at different times. The filtered supernatant and organic extracts (dissolved in 10% w/v ASB) of each *P. salmonis* strain were arranged in a 1:1 ratio with the bacterial biosensor *P. putida* EL106 (RPL4cep) (10^9^ cells/mL). Samples and controls were incubated for 24 and 48 h at 27°C. PP23: *P. putida* EL 106 (RPL4cep); Controls: *P. putida* EL106 (RPL4cep) (10^9^ cells/mL) + 250 nM C8-HSL. Emitted fluorescence was recorded in a microtiter plate reader at 515 nm. RFU, relative fluorescence units. Asterisks indicate statistical significance (ANOVA P ≤ 0.01, n = 3) between the supernatant and the organic extracts. Error bars: standard deviation.

We only detected fluorescence from the biosensor when evaluating the supernatant from Ps007 at 24 h (**Figure 3**); however, the supernatants of both strains induced fluorescence at 48 h. We detected fluorescence emission from the organic extracts of both strains at both times. In this sense, the fluorescence intensity induced by both strains was significantly higher at 48h (3864 ± 106 RFU and 3826 ± 55 RFU for *P. salmonis* LF-89 and Ps007 strains (P < 0.01), respectively) (**Figure 3**). Furthermore, this emitted fluorescence is significantly (P < 0.01) higher than that induced in the supernatants of the LF-89 and Ps007 strains; 869 ± 50 RFU and 2088 ± 50 RFU, respectively. Therefore, a significant increase in fluorescence measured was observed in the organic extracts with respect to the supernatant for each strain.

### *P. salmonis* Produces N-Acetyl-Homoserine Lactone

**Figure 4:**
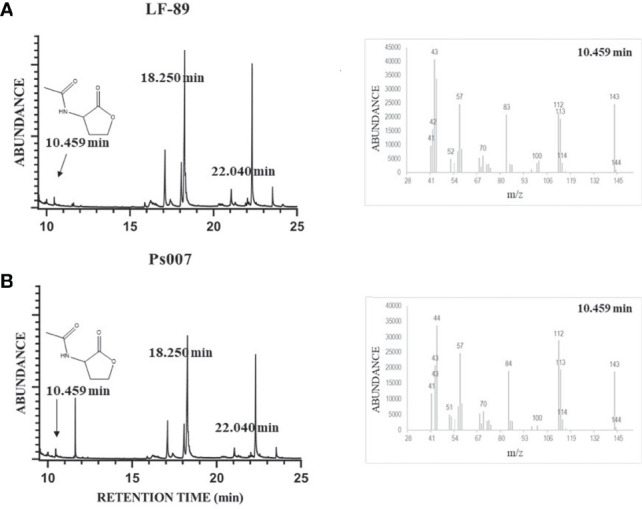
Identification of AHLs molecules in *P. salmonis* culture organic extract by GC/MS analysis. LF-89^T^ **(A)** and Ps007 **(B)** spectrum. Mass spectrum at t=10.459 min was included for both samples. The standard solutions of C4-HSL, C6-HSL, C8-HSL, and C12-HSL were prepared in GC-ECD/FID-grade acetone. MS conditions were as follows: electron ionization source set to 70 eV, MS Quad 150°C, MS Source 230°C. The mass spectrometer was run in full-scan mode (m/z 15–350) and Selected Ion Monitoring (SIM) at m/z 143.The sensor and GC/MS results are representative of three independent experiments.

Four standard solutions were assessed in GC/MS without derivatization using the full-scan acquisition mode: C4-HSL, C6-HSL, C8-HSL, and C12-HSL. We detected and identified the peaks from the respective mass spectra using the retention times of standard samples (**Table 1**). Similar chromatographic peaks were obtained from organic extracts samples from both strains (**Figures 4A, B**, respectively). However, most of the peaks did not match the retention times of the assayed standards described in **Table 1**. Additionally, the C8-HSL and C12-HSL standards matched two peaks in the samples (18.250 min and 22.040 min, respectively). However, the fragmentation pattern in scan mode showed a negligible m/z of 143. The samples were then evaluated using the SIM mode at m/z 143. The SIM chromatogram showed that neither of the possible compounds, C8-HSL or C12-HSL, generated a significant signal at m/z 143. Therefore, none of the available standards were detected in the samples. Nevertheless, the SIM chromatogram showed an intense signal in 10.459 min, where the rest of the peaks had an area at least 1,000 times lower. According to the fragmentation pattern, the compound N-acetyl-homoserine lactone (C2-HSL) (peak SIM at 10.459 min) was the most probable AHL molecule identified in both *P. salmonis* samples. Nevertheless, the SIM chromatogram showed an intense signal in 10.459 min, where the rest of the spectrum peaks had an area at least 1,000 times lower than m/z 143 (**Supplementary Figure 2**), becoming in it the molecular ion of this compound (M^·+^). According to the fragmentation pattern, the compound N-acetyl-homoserine lactone (C2-HSL; C_6_H_9_NO_3_) was the most probable AHL molecule identified in both *P. salmonis* samples (peak SIM at 10.459 min) due to the m/z fragments detected in this peak match over 80% with the m/z fragments detected for the pure acyl-HSL standards according to NIST Mass Search (**Table 1**). Additionally, an one-factor ANOVA showed that there not exist differences in the mass fragmentations distribution obtained in the samples when these were compared with the m/z distribution of the pure standards at 95% confidence level (p> 0.05).

**Table 1:** Experimental retention times and m/z fragments detected for standards AHLs molecules and samples analyzed.

Homoserine Lactone	Acronym	Molecular ion [M+]	Retention time (min)^a^	m/z fragments^b^
N-butanoyl HL	C4-HSL	171	13.657	43,57,71,100,125,143
N-hexanoyl HL	C6-HSL	199	16.060	43,57,71,83,99,125,143,156
N-octanoyl HL	C8-HSL	227	18.245	41,57,83,101,125,143,156
N-dodecanoyl HL	C12-HSL	283	22.068	43,57,69,83,102,125,143,156
Sample	LF-89	143	10.459	43,57,70,83,100,112,143
Sample	Ps007	143	10.459	44,57,70,84,100,112,143

a See experimental section for details.

b Detected fragments.

## Discussion

The predominant QS signaling pathway in Gram-negative bacteria is the LuxI/LuxR system that utilizes acyl-homoserine lactones (AHLs) signal molecules (Mok et al., 2003). Several authors described that some salmonid bacterial pathogens, such as *Edwardsiella tarda* (Morohoshi et al., 2004), *Aeromonas hydrophila*, *A. salmonicida* (Swift et al., 1997) and *Aliivibrio salmonicida* (Hansen et al., 2015), produce AHLs to induce QS-type systems. It has been identified between two and five copies of the *luxR* gene in *P. salmonis* but didn’t identify any luxI gene orthologues (Millar et al., 2018). Additionally, LuxR has been reported in the proteome of biologically active membrane vesicles in *P. salmonis* LF-89 type Strain (Oliver et al., 2017). The known diversity of signals and receptors in microbial communication is still expanding (Chen et al., 2002; Mukherjee and Bassler, 2019). LuxR solo regulators might have evolved by gene duplication and horizontal gene transfer. An increased understanding of the evolutionary roles of QS regulators would be helpful for engineering of cell–cell communication circuits in bacteria (Xu, 2020).

Bacterial biosensors have facilitated the detection of AHLs production *via* screening-based approaches (Lumjiaktase et al., 2010). Recently, Levipan et al. (2021) did not detect short- and long-chain AHLs-type molecules in two *P. salmonis* strains *via* the colorimetric agar plate-based method. This method relies on *Chromobacterium violaceum* VIR07 and CV026 biosensors strains and C6-HSL and N-hexanoyl-DL-homoserine lactone (C10-HSL) as a positive control. Fluorescence-based biosensors have advantages over other methods because the fluorescence intensity of single cells can be measured (Rai et al., 2015).

We first carried out an experimental standardization focused on the selection of the most appropriate fluorescent biosensor bacterium. Thus, *P. putida* EL106 (RPL4cep) was selected to evaluate the production of AHLs by *P. salmonis*. *P. putida* EL106 (RPL4cep) successfully emitted fluorescent signals when was cultured under the ideal conditions for *P. salmonis*. This is the first report that suggests this biosensor possesses C2-HSL sensitivity. Nevertheless, the sensor plasmid RP4cep is highly sensitive for AHLs, with acyl side chains ranging from C8 to C12 and has a wide range (C4 to C12) of detection previously reported (Lumjiaktase et al., 2010). We observed the induction of emitted fluorescence by this biosensor strain in supernatant extracts and the organic extracts of *P. salmonis* LF-89 and Ps007 strains, suggesting that *P. salmonis* produces AHLs-type molecules. We detected and identified AHLs-type molecules in both *P. salmonis* strains *via* GC/MS. The fragmentation pattern observed in each standard coincided with the fragmentation patterns previously described for AHLs molecules (Cataldi et al., 2004). All standards showed the characteristic m/z=143 as one of the main fragments. Other important detected peaks, in the available standards, were m/z 125, 101, 57, and 43, which were similar to the fragments reported by several authors for AHLs (Pearson et al., 1995; Cataldi et al., 2004; Llamas et al., 2005; Cataldi et al., 2007; Rani et al., 2011). When the *P. salmonis* samples were analyzed it was found that even when retention times of some peaks. Furthermore, even when retention times of some peaks in the *P. salmonis* samples were close to the standards, the fragmentation patterns did not completely match with those reported for AHLs, and also did not show the characteristic m/z 143 for AHLs-type molecules (Pearson et al., 1995; Levipan et al., 2021). Therefore, this suggests an absence of C4-HSL, C8-HSL, C12-HSL and also C6-HSL, which is in agreement with the previously reported by Levipan et al. (2021) in similar samples. However, this work do not show SIM chromatograms at shorter analysis times, where we have detect a significant signal at 10.459. In this work, the search of m/z 143 was performed in the entire chromatogram time length, finding that exist this small signal in scan mode at 10.459 min that showed a strong signal at SIM mode (m/z=143, **Supplementary Figure 2**). The fragmentation pattern showed that m/z 57 and 143 were two of the main detected signals, and both are typical for the identification of AHLs in *P. salmonis* (Pearson et al., 1995; Cataldi et al., 2004; Liu et al., 2019). Additionally, fragments at m/z 43, 84 and 100 were detected, all of which are close to m/z values previously reported for other AHLs (Pearson et al., 1995; Cataldi et al., 2004; Llamas et al., 2005). These outcomes are in agreement with those results reported by Levipan et al., 2021 given by none of the available standards were detected in *P. salmonis* samples. Nevertheless, at lower retention times this small signal in scan mode at 10.459 min, with a strong signal at SIM mode (**Supplementary Figure 2**), match in the fragmentation pattern according to the experimental m/z observed for the standards with a coincidence over the 80% in the most important fragments in each sample. Additionally an one-way ANOVA showed that there is not statistical differences between the distribution in the obtained main m/z fragments of each sample compared with the main m/z fragments of the pure standards at 95% confidence level (**Supplementary Table 1**). Based on these analyses, C2-HSL is AHL-type molecule present in both strains of *P. salmonis* because the fragmented ion at m/z 143 is typical of carbonyl groups having a hydrogen atom in the γ-position (**Supplementary Figure 3**). The other fragment ions, i.e., m/z 57, 71 and 43, were probably produced by two major fragmentation processes involving an inductive effect and an α-cleavage (Cataldi et al., 2004). In addition, a peak at m/z 101 was also detected, which could be explained by the α-cleavage of the N-acyclic side chain at the secondary amino group, resulting in the molecular ion formation for de-acylated homoserine lactone (**Figure 4**). Due to no peaks were detected over m/z 143, this fragment is the molecular ion corresponding to C2-HSL. However, no peaks were detected over m/z 143. Therefore, this fragment would be the molecular ion corresponding to C2-HSL. This QS signaling molecule has been identified by liquid chromatography-tandem mass spectrometry in *Gluconacetobacter xylinus* CGMCC No. 2955 and *Gluconacetobacter* sp. strain SX-1, which are acetic acid-producing Gram-negative bacteria and typical bacterial cellulose biosynthesis strains. C2-HSL has been used as a control for QS events because it belongs to a family of cell-to-cell interaction mediators in some bacteria such as *Erwinia carotovora* ATCC39048, *Paraburkholderia* sp. BSNB-0670, *Gluconacetobacter* strains, and others (Chhabra et al., 1993; Liu et al., 2019; Hoang et al., 2020).

In conclusion, this is the first study describing the production of AHL molecules by the fish bacterial pathogen *P. salmonis*. This valuable information provides a basis for further studies on the QS system of *P. salmonis* and how it is related to other processes such as pathogenicity, cell-to-cell communication, biofilm formation, antibiotic resistance, and virulence.

## Data Availability Statement

The raw data supporting the conclusions of this article will be made available by the authors, without undue reservation.

## Author Contributions

PR, DS, JV, and CO planned most of the experiments and wrote the final version of the manuscript. DS, RR, DC, JB, and CC performed some of the experiments and participated in the writing of the manuscript. AR, NR-T and HU participated in the writing of the manuscript. All authors have approved the final article.

## Funding

This work was financially supported by the Fondo Nacional de Desarrollo Científico y Tecnológico (FONDECYT) [Grant No. 11180994], Fondo de Financiamiento de Centros de Investigación (FONDAP) Interdisciplinary Center for Aquaculture Research (INCAR) [Grant No. 15110027], FONDAP Solar Energy Research Center (SERC) [Grant No. 15110019], and Vicerrectoría de Investigación, Desarrollo y Creación Artística of the Universidad Austral de Chile (VIDCA-UACh).

## Conflict of Interest

The authors declare that the research was conducted in the absence of any commercial or financial relationships that could be construed as a potential conflict of interest.

## Publisher’s Note

All claims expressed in this article are solely those of the authors and do not necessarily represent those of their affiliated organizations, or those of the publisher, the editors and the reviewers. Any product that may be evaluated in this article, or claim that may be made by its manufacturer, is not guaranteed or endorsed by the publisher.
