# Supplementary material for: Piscirickettsia salmonis Produces a N-Acetyl-L-Homoserine Lactone as a Bacterial Quorum Sensing System-Related Molecule
Source: Front Cell Infect Microbiol. 2021 Oct 25;11:755496. doi: 10.3389/fcimb.2021.755496 (PMC8573184; doi:10.3389/fcimb.2021.755496)
Supplement: Supplementary file 1 [file DataSheet_1.docx]

Supplementary Material

## Supplementary Figures

**Supplementary Figure 1.** Response of the biosensor *P. putida* EL106 (RPL4cep) to several concentrations of HSL. The bacterial biosensor was grown in presence of HSL-C8 at a concentration range from 10 nM to 1600 nM for 24 h at 120 rpm. Control was cells grown without the addition of HSL-C8 in the medium. Green fluorescence was recorded with a microtiter plate reader at 515 nm. The fluorescence measurements were corrected for autofluorescence. RFU: relative fluorescence units. Error bars: standard deviation.


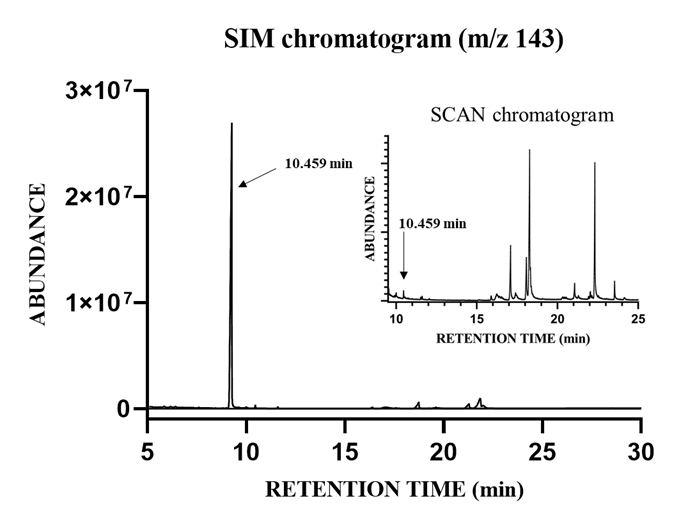


**Supplementary Figure 2.** Scan and SIM (143) chromatograms for sample LF-89^T^.


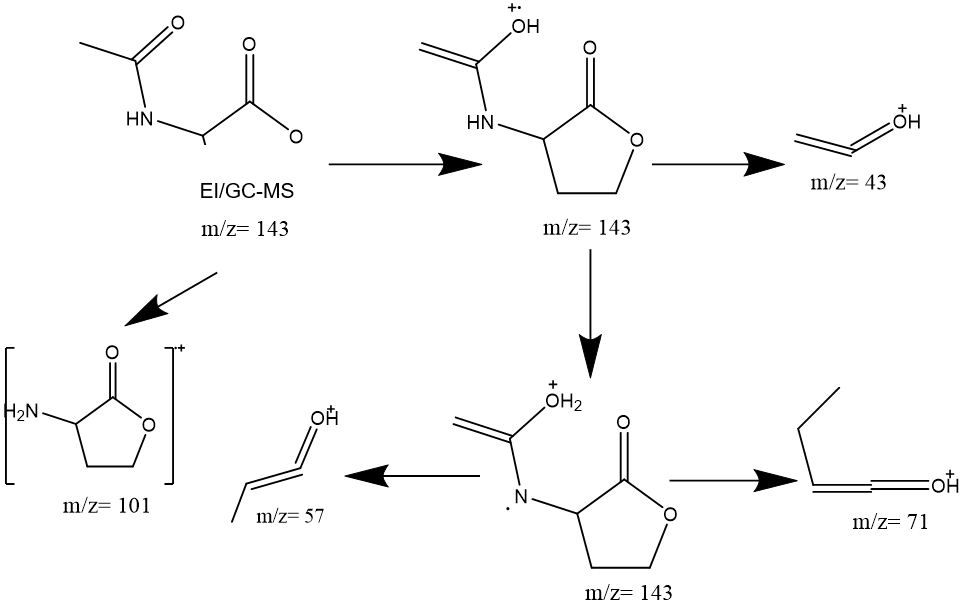


**Supplementary Figure 3.** Proposed fragmentation pattern of C2-HSL (m/z=143). Modified from Cataldi et al. (2004).

**1.2 Supplementary Tables**

**Supplementary Table 1.** Results for ANOVA one-factor.

| **ANOVA: Single Factor** |  |  |  |  |  |  |
| --- | --- | --- | --- | --- | --- | --- |
|  |  |  |  |  |  |  |
| SUMMARY |  |  |  |  |  |  |
| *Grups* | *Count* | *Sum* | *Average* | *Variance* |  |  |
| LF89 | 8 | 721 | 90.125 | 1095.55357 |  |  |
| Psal007 | 8 | 723 | 90.375 | 1080.26786 |  |  |
| C4-HSL | 6 | 539 | 89.833 | 1558.56667 |  |  |
| C6-HSL | 7 | 621 | 88.714 | 1301.90476 |  |  |
| C8-HSL | 7 | 621 | 88.714 | 1340.57143 |  |  |
| C12-HSL | 7 | 622 | 88.857 | 1326.14286 |  |  |
|  |  |  |  |  |  |  |
|  |  |  |  |  |  |  |
| ANOVA |  |  |  |  |  |  |
| *Source of variation* | *$ $* | *df* | *M$* | *F* | *P-value* | *F crit* |
| Between Groups | 21.40006 | 5 | 4.28001 | 0.00338122 | 0.99999783 | 2.46964965 |
| Within Groups | 46835.29762 | 37 | 1265.81885 |  |  |  |
|  |  |  |  |  |  |  |
| Total | 46856.69767 | 42 |  |  |  |  |
